# Metabolic fingerprinting of joint tissue of collagen-induced arthritis (CIA) rat: In vitro, high resolution NMR (nuclear magnetic resonance) spectroscopy based analysis

**DOI:** 10.17179/excli2017-938

**Published:** 2018-03-19

**Authors:** Niraj Kumar Srivastava, Shikha Sharma, Rajkumar Sharma, Neeraj Sinha, Sudhir Kumar Mandal, Deepak Sharma

**Affiliations:** 1 Neurobiology Laboratory, School of Life Sciences, Jawaharlal Nehru University, New Delhi-110067; 2 Center of Biomedical Research, Sanjay Gandhi Postgraduate Institute of Medical Sciences Campus, Lucknow-226014, India

**Keywords:** collagen-induced arthritis, extraction, metabolites, metabolism, NMR spectroscopy, oxidative stress

## Abstract

Rheumatoid arthritis (RA) is a systemic autoimmune disease whose major characteristics persistent joint inflammation that results in joint destruction and failure of the function. Collagen-induced arthritis (CIA) rat is an autoimmune disease model and in many ways shares features with RA. The CIA is associated with systemic manifestations, including alterations in the metabolism. Nuclear magnetic resonance (NMR) spectroscopy-based metabolomics has been successfully applied to the perchloric acid extract of the joint tissue of CIA rat and control rat for the analysis of aqueous metabolites. GPC (Glycerophosphocholine), carnitine, acetate, and creatinine were important discriminators of CIA rats as compared to control rats. Level of lactate (significance; p = 0.004), alanine (p = 0.025), BCA (Branched-chain amino acids) (p = 0.006) and creatinine (p = 0.023) was significantly higher in CIA rats as compared to control rats. Choline (p = 0.038) and GPC (p = 0.009) were significantly reduced in CIA rats as compared to control rats. Choline to GPC correlation was good and negative (Pearson correlation = -0.63) for CIA rats as well as for control rats (Pearson correlation = -0.79). All these analyses collectively considered as metabolic fingerprinting of the joint tissue of CIA rat as compared to control rat. The metabolic fingerprinting of joint tissue of CIA rats was different as compared to control rats. The metabolic fingerprinting reflects inflammatory disease activity in CIA rats with synovitis, demonstrating that underlying inflammatory process drives significant changes in metabolism that can be measured in the joint tissue. Therefore, the outcome of this study may be helpful for understanding the mechanism of metabolic processes in RA. This may be also helpful for the development of advanced diagnostic methods and therapy for RA.

## Abbreviations

NMR: Nuclear Magnetic Resonance; RA: Rheumatoid arthritis; CIA: Collagen-induced arthritis; BCA: Branched chain amino acids; GPC: Glycerophosphocholine; SOD: Superoxide dismutase; GPx: Glutathione peroxidase; CAT: Catalase.

## Introduction

Collagen-induced arthritis (CIA) in rats is an autoimmune disease model and in many ways shares features with clinical rheumatoid arthritis (RA) (Kannan et al., 2005[22]). Therefore, the CIA rat model is considered as a relevant animal model of RA and has been used in many studies to investigate the pathogenesis of inflammatory arthritis (Feldmann et al., 1996[14]; Holmdahl et al., 1989[20]; Trentham, 1982[56]), and also to evaluate the pharmaco-therapeutic potential of anti-arthritic medications (Salvemini et al., 2001[42]; Kumar et al., 2009[25]; Patro et al., 2011[38]). RA is an autoimmune disease with a global prevalence of about 1 % (Lee and Weinblatt, 2001[27]). Despite of the best efforts made in defining its etiology and pathogenesis, the approach is still in its infancy (Feldmann, 2001[13]; Firestein, 2003[15]). This disease is characterized by chronic inflammation of the synovial joints and destruction of articular cartilage (Araújo et al., 2015[1]). There are a number of proposed causes for RA which includes genetic predisposition, pathogenetic immune-inflammatory responses triggered by environmental agents, autoimmunity directed against components of synovium and cartilage, dysregulated production of cytokines, recruitment of immune-inflammatory cells through induction of inflammatory cell and transformation of synovial cell into autonomously proliferating cells (Kinne et al., 2000[23]; Fassbender et al., 1983[12]). All these causes collectively indicate the cytokine-mediated inflammatory processes in RA and have a strong impact on metabolism. The degree of metabolic alterations and the types of metabolites may have excellent markers of cytokine-mediated inflammatory processes in RA (Cederholm et al., 1997[6]; Kotler, 2000[24]; Brindle et al., 2002[4]). Therefore, it is of paramount importance to investigate the metabolic changes and to find out the targeted markers. Here in this study, we have used CIA rat as a model system for the investigation. Metabolomics is an important part of systems biology and usually defined as 'quantitative measurements of dynamic multi-parametric metabolic responses of living systems to pathological stimuli or genetic modifications' (Nicholson et al., 1999[34]). NMR (Nuclear Magnetic Resonance) spectroscopy based metabolomics is a potential tool for the analysis of metabolites in biofluids, such as plasma, serum, CSF (cerebrospinal fluid), pus, saliva, cervicovaginal secretions and urine, and tissue extract (Gebregiworgis and Powers, 2012[16]). NMR is a non-destructive and rapid technique which requires minimum sample processing. This property makes it the most efficient method for qualitative as well as quantitative analysis of metabolites with excellent repeatability and reproducibility (Gebregiworgis and Powers, 2012[16]). NMR spectroscopy is successfully applied to metabolic profiling of various diseases such as inflammatory bowel disease (Marchesi et al., 2007[28]), ocular inflammatory disease (Young et al., 2009[61]), neurological diseases (Sinclair et al., 2010[47]), coronary heart disease (Brindle et al., 2002[4]) and RA (Naughton et al., 1993[32]). Synovial fluid of patients with RA showed an elevated level of lactate, lipoprotein-associated fatty acids and acute-phase glycoproteins as compared to healthy subjects (Naughton et al., 1993[32]). Metabolic status of synovial fluids as compared to serum from patients with RA has shown markedly elevated lactate, ketone bodies and diminished glucose and lipid levels (Naughton et al., 1993[32]) which are very useful for differentiating between patients with RA and osteoarthritis (OA) (Meshitsuka et al., 1999[30]). Previous studies have shown that septic and non-septic RA could also be differentiated by the analysis of lactate level in the synovial fluid (Gobelet and Gerster, 1984[17]). The serum metabolic fingerprinting in patients with RA is clearly distinct from that of healthy controls. Studies have revealed that lactate, histidine, and lipid levels are significantly different in patients with RA as compared to healthy controls (Young et al., 2013[60]; Sitton et al., 1987[49]). Synovial fluid and serum were used for the analysis of metabolites in patients with RA (Naughton et al., 1993[32][33]; Meshitsuka et al., 1999[30]; Gobelet and Gerster, 1984[17]; Young et al., 2013[60]; Sitton et al., 1987[49]). Similarly, metabolomics analysis was also performed on serum/plasma and urine samples of CIA rats (Ding et al., 2014[11]; Zhang et al., 2014[63]; Yue et al., 2013[62]). Lipid components of joint tissue of CIA rats were analyzed by NMR spectroscopy (Srivastava et al., 2014[51]). To the best of our knowledge, there are no scientific reports about the NMR spectroscopy based analysis of aqueous metabolites in joint tissue of CIA rat. In the present study, assignment and selective quantification of metabolites in joint tissue of CIA rats as compared to control rats were performed by NMR spectroscopy. 

## Materials and Methods

### Animals

Female Wistar rats of 6-8 weeks (weight 130-150 gm) were taken for the induction of arthritis. All these rats were issued and maintained at the Central animal facility of Jawaharlal Nehru University, New Delhi (India). These experimental rats were housed separately in cages, fed with standard rodent chow (Hindustan Lever Limited, India) and water *ad libitum*. Standard parameters were taken into care during their housing and whole experimental phase. These were photoperiods, darkness, environmental temperature, and humidity. Photoperiods equalled 12 h of light and 12 h of darkness daily, with the environmental temperature and humidity maintained at 25 °C + 2 and 42 % + 5, respectively. All experimental protocols were approved by the Committee for the Purpose of Control and Supervision of Experimental Animals (CPCSEA) and the Institutional Animal Ethical Committee (IAEC) of Jawaharlal Nehru University, New Delhi, India.

### Chemicals

All the chemicals were purchased from Sigma Aldrich Chemical Co. (St. Louis, MO). Antibodies of TNF-α, IL-1β and IL-6 were purchased from Peprotech Inc., USA. All other chemicals and solvents used were of analytical grade.

### Induction of arthritis

These experimental female Wistar rats were randomly allocated into the two groups of five animals each (a) Group I: Rats served as healthy 'control' with no collagen immunization, (b) Group II: Rats immunized with collagen designated as "CIA". 

The CIA was induced as previously described by Srivastava et al. (2014[51]). Briefly, porcine immunization-grade native collagen type II (Chondrex) was dissolved in 0.05 mol/l acetic acid at a concentration of 2 mg/ml by stirring overnight at 4 °C. The emulsion was prepared by homogenizing collagen type II with Freund's complete adjuvant (1:1) at 4 °C. Each rat was immunized with 200 μg of emulsified collagen type II, intradermally at multiple sites on the back and the base of the tail followed by a booster dose with the same antigen preparation on day 7. Arthritis was allowed to develop for 28 days.

### Macroscopic scoring of CIA

In all the experimental rats, the clinical scoring of CIA rats was performed. Clinical score was based on the composite index of disease severity and the number of affected limbs. The clinical score was assessed every 4 days to monitor disease progression as described previously (Cuzzocrea et al., 2000[9]). Since the CIA is more pronounced in the hind-limbs, so, the scoring of rat hind-limbs only has been taken into account to assess the severity. Two independent observers, blinded to the experimental groups, performed the scoring.

### Paw diameter measurement and Arthritic Index (AI)

In all the experimental rats, the hind paw edema was measured every 4^th^ day throughout the experiment using Vernier callipers. The arthritic indices (AI) were calculated as described in the previous report (Coelho et al., 2004[7]) using the formula:


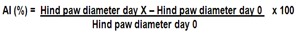


### Body weight examination

The body weight was measured every 4^th^ day in all the experimental rats. The measurement of body weight was performed according to our previous study (Srivastava et al., 2014[51]). Variation in body weight (%) of each individual animal after the onset of arthritis on day 12 was calculated using the formula below.

### Radiological evaluation of CIA

On day 28, all the experimental rats were sacrificed. Hind limbs were removed and right hind leg was fixed at 10 % buffered formalin for radiological evaluation. All radiographs were taken with X-ray film (Kodak Diagnostic Film) using MBR-1505R (Hitachi Medical Corporation, Tokyo, Japan). Settings for radiography were 5 mA, 40 kV and 1 s exposure. Films were placed 60 cm below the X-ray source.

### Assessment of arthritic damage through histology

Arthritic damage was also examined through histology. The knee joint was excised from the fixed hind leg, its skin and muscular parts were trimmed off and subjected to decalcification in 5 % nitric acid for 7-10 days, processed for paraffin embedding, sectioned at 8 μ followed by staining with hematoxylin-eosin and studied using light microscopy.

### Biochemical analysis [Determination of in vivo enzymatic activity and lipid peroxidation] 

The activity of antioxidant enzymes SOD (superoxide dismutase), CAT (catalase), GPx (glutathione peroxidase) and LP (lipid peroxidation) were measured in joint tissue of CIA rats and control rats by applying the procedure of Singh et al. (2000[48]). The LP was estimated in joint tissue of CIA rats and control rats by the method of Ohkawa et al. (1979[35]).

### Preparation of joint tissue homogenate for the determination of in vivo enzymatic activity and lipid peroxidation

For the preparation of joint tissue homogenate, the joint tissues were removed, rinsed quickly in cold saline solution and homogenized in 0.05 M Tris-HCl buffer (*p*H =7.4) to yield a 5 % (w/v) homogenate. The homogenate was centrifuged at 1000 g for 10 min. The resulting supernatant was transferred into pre-cooled centrifugation tubes and centrifuged at 12,000 g for 30 min. The supernatant (containing the cytosolic fraction) was diluted appropriately followed by the removal of residual lipid layers. It was then used to estimate the total cytosolic SOD, CAT, GPx and LP. The homogenate was stored at -80°C till biochemical analysis.

### Determination of SOD, GPx and CAT activity 

The activity of total cytosolic SOD, GPx and CAT enzymes in joint tissue homogenate were determined and expressed as units/mg protein (Singh et al., 2000[48]).


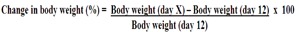


### Estimation of LP

Thiobarbituric acid-reactive substance (TBARS), an index of lipid peroxidation in joint tissue homogenate was estimated by the method of Ohkawa et al. (1979[35]). The amount of TBARS was determined spectrophotometrically at 532 nm and expressed as nanomoles of malondialdehyde (MDA)/mg protein. Tetramethoxypropane (TMP) was used as a standard.

### Measurement of cytokines in joints

Cytokine measurements were also performed on joints of all the experimental rats. For this purpose, whole joints were powdered with liquid nitrogen by using a pestle-mortar. Powdered tissue was weighed and homogenized (1:5 w/v) in 0.02 M Tris-HCl buffer ( *p*H =7.4) containing 20 mM MgCl_2_, 1 mM PMSF, 10 μg leupeptin and aprotinin, 1 mM ATP, 5 mM EDTA, 50 mM DTT and 3 μl protease inhibitor cocktail per 10 ml buffer. Homogenate was first centrifuged at 4000 rpm, 4°C for 15 min to remove the debris and bony portions. The collected supernatant was further centrifuged at 16,000 rpm for 15 min and the resultant clear supernatant was aliquoted and stored at -80 °C for estimation of cytokines. Concentrations of plasma and joint cytokines, TNF-α, IL1- β and IL-6 were measured by indirect ELISA.

Plates (Nunc) were coated with 10 μg proteins/well diluted in carbonate buffer at 37 °C for 1-2 h. The plates were washed thrice with PBST (0.2 % Tween 20 in 1X PBS) and blocked using blocking buffer (1 % skimmed milk in 1X PBS) for 1 h at 37°C. After washing, 100 μl/well anti-rat polyclonal antibodies against TNF-α, IL1-β and IL-6 were added and incubated at 37 °C for 1 h. This was followed by washing the wells and then 100 μl/well (1:1000 diluted) anti-rabbit HRP labelled IgG secondary antibody was added. After incubation followed by washing, developing buffer (10 mg/ml orthophenylene diamine dissolved in 150 mM citrate-phosphate buffer, *p*H 5.6 with 30 % H_2_O_2_) was added. The plates were incubated for 5-10 min at RT (room temperature), the reaction was stopped with the stop solution (2 N H_2_SO_4_) and the plate was read at 490 nm using Spectra Max Plus from Molecular Devices using the SoftMax-Pro software. Bovine serum albumin, at a concentration of 5 μg/ml (100 μl/well) was included as a negative control in each assay performed and the wells incubated with coating buffer instead of plasma were used as the blank.

The concentration of cytokines was expressed in pg/ml. Each sample was measured in triplicates and values were derived from the calibration curve.

### Perchloric acid extraction of joint tissue

Aqueous metabolites were extracted from joint tissue of the CIA and control rats by the method of perchloric acid extraction, as described previously (Srivastava et al., 2008[50]). After completing the extraction, the sample was lyophilized. All samples were re-dissolved in 0.5 ml deuterated water (pH = 7.0) and taken in 5 mm NMR tube (Wilmad no. 528, USA). The ^1^H-NMR experiments were performed on a Bruker Avance 400 MHz spectrometer (Bruker Biospin, Zurich, Switzerland) at 25 °C temperature. 

### ^1^H NMR spectroscopy parameters

One-dimensional proton NMR experiments, using single pulse sequence were performed. Spectral width used was 8000 Hz with time domain data points of 32 K. Flip angle of the radio frequency pulse was 90° with a complete relaxation delay of 7 seconds (relaxation delay plus acquisition time) to ensure complete recovery of magnetization to the equilibrium between the scans. Typically, 128 scans were accumulated for each sample and the resultant data were Fourier transformed after multiplying by exponential window function using a line broadening of 0.34 Hz. Sealed and reusable glass capillary with a known concentration of TSP [3-(trimethylsilyl) propionic-2, 2, 3, 3-d4 acid, sodium salt] in D_2_O was used as a standard reference. The NMR spectra of all the samples were recorded under uniform conditions.

### Assignment of the metabolites in the perchloric acid extraction of joint tissue

Assignments of the metabolites in the perchloric acid extract of joint tissue of CIA rats as well as control rats were completed with the help of two-dimensional (2D) double quantum filtered correlation spectroscopy (DQF-COSY) experiment. The assignments were confirmed by literature (Zhong et al., 2012[64]; Shao et al., 2014[43]; Atherton et al., 2006[2]; Hwang et al., 2010[21]; Sharma et al, 2003[45][46]). Spectra of standard components were recorded individually as well as in a mixture with an appropriate concentration. Protons of amino acids are *p*H sensitive, the resonances from glutamate, glutamine and alanine were confirmed by recording the spectra of these compounds in the *p*H range 7-9. The N^+^(CH_3_)_3_ resonances of GPC (Glycerophosphocholine) and carnitine were identified by carrying out the experiment on the mixture of these two compounds. 

### Quantification of metabolites

Quantification of metabolites was performed by using a computer program, which is devised for such purposes and also used in the previous studies (Srivastava et al., 2008[50], 2014[51]). Metabolite concentrations were calculated in the mmol/mg weight of the joint tissue.

### Statistical analysis

The mean level of selected metabolites in the perchloric acid extraction of the joint tissue of the CIA rats and control rats was compared by student “t” test for two independent groups. The *p*-value less than 0.05 was considered to be significant. Correlation and DFA (discriminant function analysis) were also performed. The data management and analysis were carried out using statistical software SPSS version 15.0.

## Results

### Parameters for establishment of arthritis in rats (CIA rats)

The outcome of characteristic parameters of CIA rats as compared to control rats are described in the following way:

### Severity of CIA in Wistar rats

**Figure 1:**
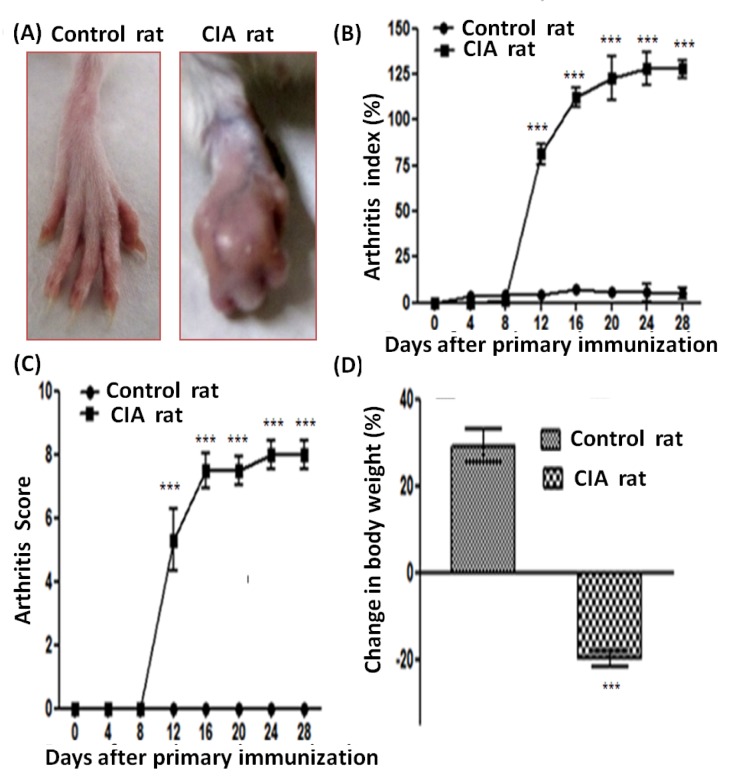
Representative photographs of (A) hind paws of control and CIA rats, effect of immunization with type II collagen on arthritis progression as measured through, (B) arthritis index, (C) arthritis score and (D) change in body weight [values are represented as mean + SD of 5 animals per group, ***p < 0.0001].

Collagen induced arthritis was developed in the experimental rats immunized with type II collagen as shown in Figure 1(A)(Fig. 1). Clinical presentation of arthritis (mild periarticular erythema and edema) exposed by the redness and swelling of the joints first appeared in the hind paws approximately 10 days post challenge. Progression of disease was enhanced in frequency and severity in a time-dependent manner and maintained a plateau of the peak of CIA response from day 15-30 (mean AI of 125.01) [Figure 1(B)(Fig. 1)]. There was no enlargement appearing in hind paw diameter over time. Hind paw swelling and erythema also presented a time-dependent enhancement in severity with maximum arthritis scores of ~8 observed between days 12 and 30 in rats immunized with CII [Figure 1(C)(Fig. 1)]. There was no macroscopic confirmation of the hind paw swelling and erythema in the control rats.

### Change in body weight

The body weight of all the experimental rats was recorded for every 4^th^ day during the whole duration of the experiment. The onset stage of arthritis was connected with severe body weight loss that continued till the end of experimental duration, i.e., day 30 in the CIA rats [Figure 1(D)(Fig. 1)]. Control rats showed a gradual increase in their body weight with respect to time.

### Radiographic and histopathological analysis

**Figure 2:**
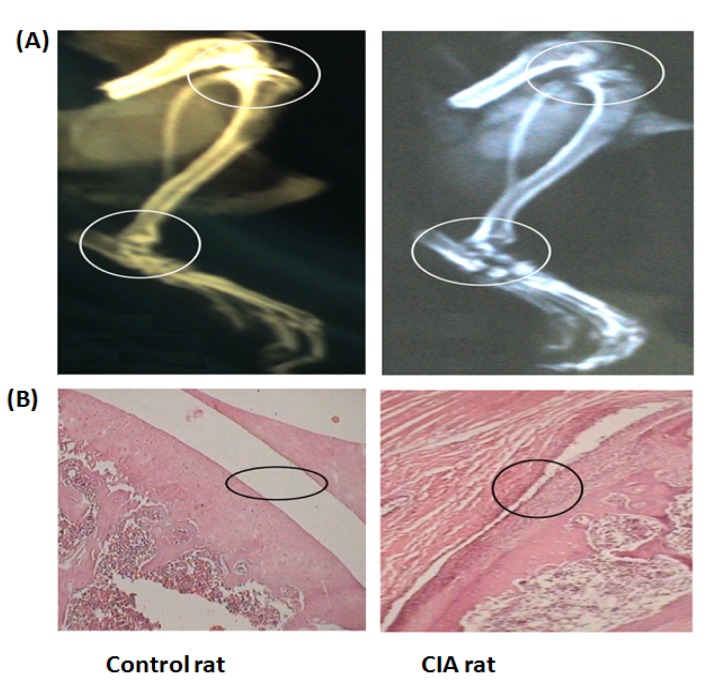
(A) Comparative radiographical representation of the hind limbs (showing the tibiotarsal and tibiofemoral joints with circles) of control and CIA rats. No pathological changes were observed in the tibiotarsal joints of control rats; CIA rats suffered from bone resorption and joint narrowing, (B) Comparative representation of histological photographs (hematoxylin and eosin stained slides) of knee joints of control and CIA rats. The circles indicate, smooth and monolayer synovial cell lining of control rats, hyperplastic synovial cells, erosion, and disruption of synovial lining in CIA group.

Radiographic analysis of the hind paws from CIA rats presented soft tissue swelling, bone matrix resorption and joint space narrowing on day 30 of the experiment [Figure 2(A)(Fig. 2)]. There was no radiographical evidence of arthritic features in the control rats. After finishing the experiment on day 30, histological evaluation of the joints of arthritic animals presented a distinct characteristic feature of severe arthritis like synovial hyperplasia, massive mixed infiltration (neutrophils, macrophages and lymphocytes) [Figure 2(B)(Fig. 2)] along with articular cartilage and bone erosion. There was no proof of arthritic features of inflammation and tissue destruction in the joints of control rats. 

### Estimation of LP

**Figure 3:**
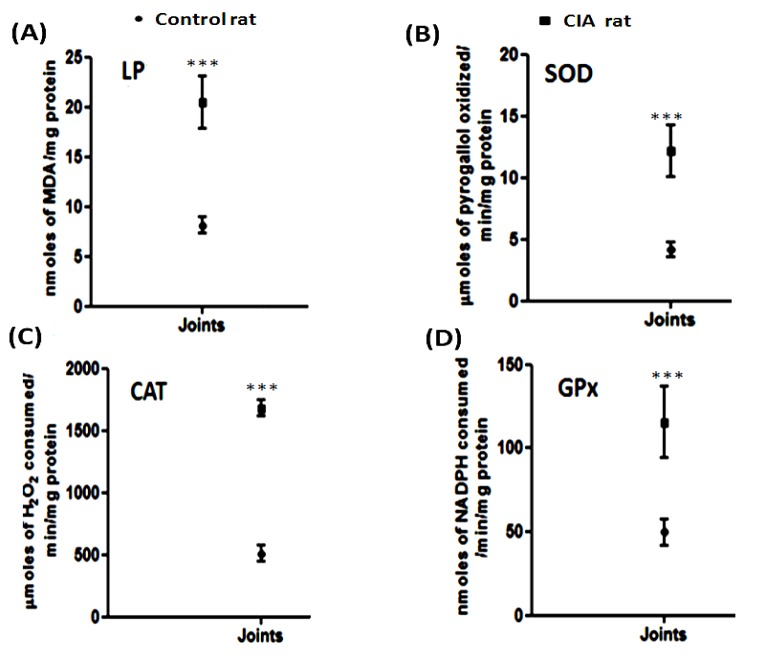
Comparative graphical representation of (A) LP (Lipid peroxidation) estimation, and activity of antioxidant enzymes (B) SOD, (C) CAT, (D) GPx in joints of control and CIA rats [values are represented as mean + SD of 5 animals per group, ***p < 0.0001].

Level of lipid peroxidation was found to be higher in CIA rats as compared to control rats [Figure 3(A)(Fig. 3)]. The lipid peroxide levels were increased by 2.84 fold (p < 0.0001) in the joints of CIA rats as compared to control rats. The results showed that the joints were much affected and are vulnerable to peroxidation due to increased oxidative stress in the CIA.

### Estimation of enzyme activity

SOD, GPx and CAT activities were determined in the joints of CIA rats and control rats [Figure 3(B-D)(Fig. 3)]. SOD, GPx and CAT activities were found to be higher (p < 0.0001) in the CIA rats as compared to the control rats. CAT showed the maximum increase of 2.87 fold in the arthritic rat joints followed by rising of 2.22 and 1.65 fold in the activity of SOD and GPx, respectively.

### Measurement of cytokines in joints

**Figure 4:**
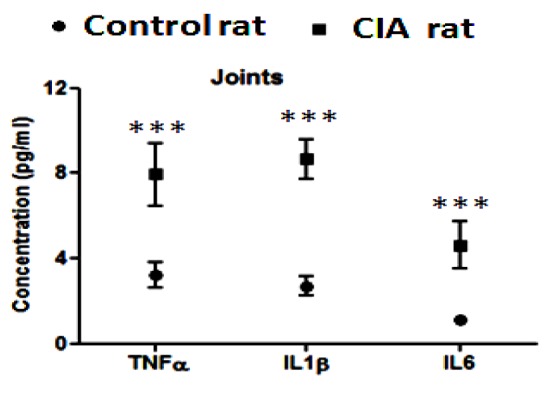
Comparative graphical representation of the cytokine level (TNF-α, IL-1β and IL-6) measurement of joint tissue of control and CIA rats [values are represented as mean + SD of 5 animals per group, ***p < 0.0001].

Levels of TNF-α, IL-1β and IL-6 were found to be higher (p < 0.0001) in joint tissue of CIA rats as compared to control rats (Figure 4(Fig. 4)).

### Assignment of metabolites in the perchloric acid extraction of the joint tissue

**Figure 5:**
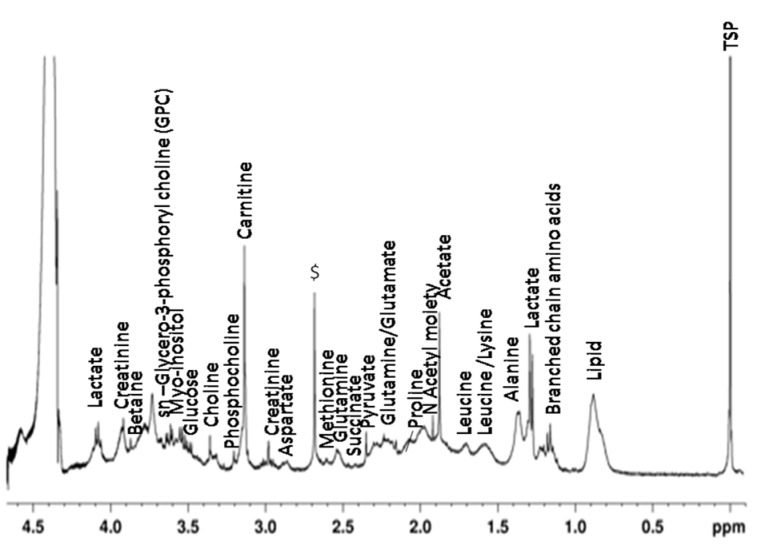
^1^H NMR spectrum of aqueous extract (perchloric acid) of joint tissue of CIA as well as control rat with assigned metabolites ($ = unassigned component and TSP = reference).

Assignments of various metabolites in the perchloric acid extract of the joint tissue of the CIA rats as well as control rats were represented in the Figure 5(Fig. 5).

### Quantification of metabolites in the perchloric acid extraction of the joint tissue

**Figure 6:**
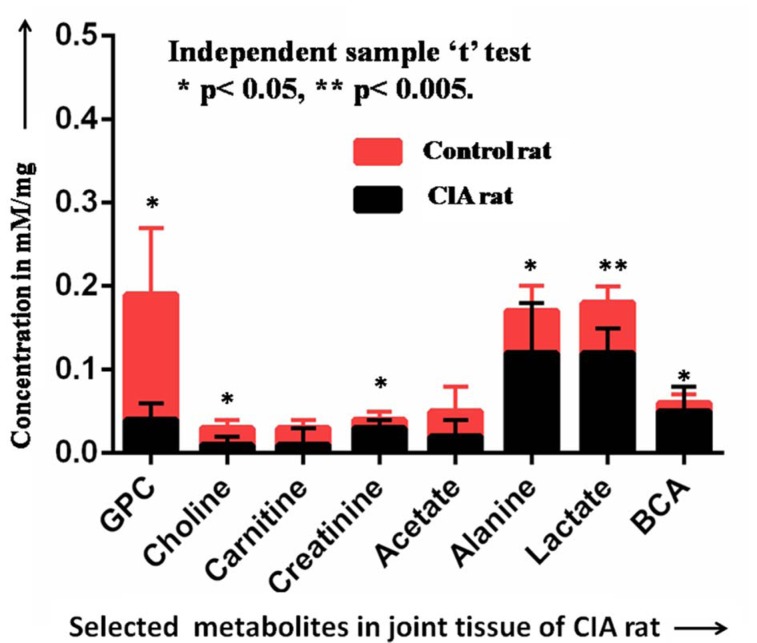
Quantitative comparison of selected metabolites (GPC, choline, carnitine, acetate, alanine, lactate and BCA) in between CIA and control rats [Concentration are expressed (mean + SD) in mM/ mg; Statistical significant difference: *p < 0.05, **p < 0.005].

Quantification of BCA (Branched chain amino acids: valine, leucine and isoleucine), lactate, alanine, acetate, carnitine, choline, GPC and creatinine was performed in the perchloric acid extract of joint tissue of CIA rats as well as control rats. Level of acetate was reduced in CIA rats as compared to control rats, but independent sample “t” test showed no significant difference (*p *= 0.78). Similarly, carnitine level was also reduced in CIA rats as compared to control rats, but the statistically significant difference is not observed (*p *= 0.54). Lactate, alanine, BCA and creatinine levels were higher in CIA rats as compared to control rats. There is a significant difference observed between lactate (*p *= 0.004), alanine (*p *= 0.025), BCA (*p *= 0.006) and creatinine levels (*p *= 0.023). Level of choline and GPC were reduced in CIA rats as compared to control rats. Choline (*p *= 0.038) and GPC (*p *= 0.009) were statistically significant in CIA rats as compared to control rats. Statistical analysis represented the quantitative difference for metabolites in CIA rats as compared to control rats (Figure 6(Fig. 6)).

Correlation in between alanine and lactate was poor (Pearson correlation = 0.34) for CIA rats, but no correlation was observed in control rats. The coefficient of correlation in between carnitine and BCA was somehow better (Pearson correlation = 0.44) than alanine to lactate correlation for CIA rats. Correlation in between choline and GPC was good and negative (Pearson correlation = -0.63) for CIA rats as well as for control rats (Pearson correlation = -0.79).

DFA analysis was performed for the discrimination or classification (100 %) of CIA rats and control rats on the basis of two parameters (choline and GPC). The weightage for choline and GPC was 142.18 and 25.92, respectively. Constant value for the DFA equation was -4.755 and cut off value was zero.

## Discussion

All the earlier reports only described the metabolites of serum/plasma, synovial fluid and urine of patients with RA or animal model of RA (Young et al., 2013[60]; Sitton et al., 1987[49]; Ding et al., 2014[11]; Zhang et al., 2014[63]; Yue et al., 2013[62]). There were no reports with reference to the metabolites of joint tissue of the animal model of RA. Qualitative and quantitative analysis of metabolites is collectively considered as metabolic fingerprinting of the joint tissue of CIA rat as compared to control rat. The metabolic fingerprinting of joint tissue of CIA rats as compared to control rats is presented. Proton NMR spectroscopy based metabolomics analyses were utilized to explore the metabolic differences between the CIA rats and control rats.

BCA (Branched chain amino acids: valine, leucine and isoleucine) are significantly higher in the CIA rats as compared to the control rats. Accumulation of free amino acids in plasma of patients with rheumatoid arthritis as compared to healthy subjects was already reported (Borden et al., 1950[3]). Rise in the level of BCA in plasma of patients with rheumatoid arthritis as compared to healthy control also existed in the literature (Trang et al., 1985[55]). Breaking of joint, muscle tissue protein observed during the inflammatory process. Inflammation induced damage in arthritic condition (St. Clair et al., 2004[52]) is responsible for the accumulation of BCA in joint tissue. BCA is playing a vital role in the generation and preservation of muscle tissue through protein synthesis (Casperson et al., 2012[5]; Qin et al., 2011[39]). In this regard, higher demands of it, in joint areas, which is further responsible for the mobilization of these amino acids via blood to the joint tissue. A hallmark of RA pathology is the remarkable increase of cellularity accompanied by angiogenesis in the synovial membrane. This reflects the increase of cellular recruitment from the circulation, and probably also an increased retention of cells in the synovial membrane (Henderson et al., 1995[19]). This event is also required free amino acids, i.e. BCA.

Carnitine level is decreased in CIA rats as compared to control rats, but does not show the significant difference and also showed the poor correlation with BCA. This indicates the somehow poor catabolism of branched chain fatty acids, because these are produced by transamination reactions of branched chain amino acids (Rajendram et al., 2015[40]; Meister, 2012[29]). Carnitine is an essential component of fat metabolism and performed the role to transport fatty acids into the mitochondria for degradation or catabolic process (Reavley, 1998[41]). 

Level of acetate also decreased in CIA rats, without showing the significant difference as compared to control rats, but it supported the decreased level of catabolism of fatty acids in joint tissue of CIA rats because the ultimate product of the fatty acids is acetate (Nath, 2007[31]). 

Lactate and alanine are significantly higher in joint tissue of CIA rats as compared to control rats. Rise in lactate level is an indicator of inflammation and enhancement of the rate of anaerobic glucose catabolism (Naughton et al., 1993[32][33]) in joint tissue of CIA rats. A higher level of lactate also found in synovial fluid and serum of patients with RA (Naughton et al., 1993[33][34]). LDH (Lactate dehydrogenase) activity was also higher in the joint tissue of CIA rats (Sharma et al., 2011[44]). In this way, the hypoxic and acidic conditions are developed in the synovium of joint tissue of CIA rat (Naughton et al., 1993[32][33]). The higher amount of accumulated lactate is converted into alanine via transamination process (Meister, 2012[29]). Alanine is also required during angiogenesis process of joint tissue (Henderson et al., 1995[19]) and taken from blood. These events are responsible for the rising of alanine in joint tissue of CIA rats.

Level of choline and GPC are reduced in joint tissue of CIA rats as compared to control rats. These two components are completely discriminating the CIA rats as compared to control rats in DFA analysis. Choline and GPC are catabolic product of phosphatidylcholine. Under the process of catabolism, the phosphatidylcholine is converted into GPC and then choline (Vance, 1989[57]). Negative and good correlation between choline and GPC in joint tissue of the CIA rats and control rats showed the catabolism of phosphatidylcholine. The rate of catabolism of phosphatidylcholine is decreased in CIA rat as compared to control rat because the Pearson correlation (-0.63) for CIA rats is less as compared to control rats (Pearson correlation = -0.79). Various studies have shown that the amount of phospholipids increases in joint tissue (Yue et al., 2013[62]; Hawthorne and Ansell, 1982[18]; Lazarevic et al., 1992[26]; Vijayakumar et al., 2005[58]; Weljie et al., 2007[59]; Pasupathi et al., 2009[37]; Park et al., 1999[36]), which further supported the decline in the level of choline and GPC. Choline is utilized for the synthesis of phosphatidylcholine (Vance, 1989[57]). Phosphatidylcholine is most important phospholipids and required for the synthesis of cell membrane (Hawthorne and Ansell, 1982[18]). This phosphatidylcholine may be utilized for the regenerative process in CIA rats.

Creatinine is significantly increased in the joint tissue of CIA rats as compared to control rats. Phosphocreatine is an important component of muscle energy metabolism (Conway and Clark, 1996[8]; Tortora and Derrickson, 2011[54]). Both creatine and creatine phosphate get converted into creatinine by a non-enzymatic, irreversible and spontaneous reaction (Talwar and Srivastava, 2006[53]). Damage of joint muscle is a critical event in CIA rats (Henderson et al., 1995[19]) and may be most of the creatine phosphate converted into creatine, which is further converted into creatinine (Talwar and Srivastava, 2006[53]). The higher amount of creatine is accumulated in the joint tissue. Creatinine is a waste product and excreted in urine (Talwar and Srivastava, 2006[53]). Creatinuria is a pathological condition found in patients with rheumatoid arthritis and osteoarthritis (Dawson and Salt, 1952[10]), which is also, supporting the higher level of creatinine in joint tissue. 

## Conclusion

In conclusion, the metabolic profile of CIA rats as compared to control rats reflects inflammatory activity and inflammation-induced damage. These metabolites selectively separated the CIA condition as compared to control stage and also suggest that the underlying inflammatory processes drive significant changes in metabolism in CIA rats. It may give an additional approach to the mechanism of inflammation in arthritis to explain to some extent. The outcome of this study may be useful for the development of advanced diagnostic methods and therapy for RA.

## Notes

Shikha Sharma and Rajkumar Sharma contributed equally as second author.

## Acknowledgements

Authors are thankful to the University Grant Commision (Dr. DS Kothari Fellowship), Government of India, New Delhi for providing the financial assistance to complete this research work [UGC grant number: No.F.4-2/2006 (BSR)/13-194/ 2008 (BSR)].

## Conflict of interest

The authors declare that there is no conflict of interest in any form related to the research work.

## Compliance with ethical requirements

All experimental protocols were approved by the Committee for the Purpose of Control and Supervision of Experimental Animals (CPCSEA) and the Institutional Animal Ethical Committee (IAEC) of Jawaharlal Nehru University, New Delhi, India. 

## References

[1] Araújo VM, Melo IM, Lima V (2015). Relationship between periodontitis and rheumatoid arthritis: Review of the literature. Mediators Inflamm.

[2] Atherton HJ, Bailey NJ, Zhang W, Taylor J, Major H, Shockcor J (2006). A combined 1H-NMR spectroscopy- and mass spectrometry-based metabolomic study of the PPAR-alpha null mutant mouse defines profound systemic changes in metabolism linked to the metabolic syndrome. Physiol Genomics.

[3] Borden AL, Wallraff EB, Brodie EC, Holbrook WP, Hill DF, Stephens CA (1950). Plasma levels of free amino acids in normal subjects compared with patients with rheumatoid arthritis. Proc Soc Exp Biol Med.

[4] Brindle JT, Antti H, Holmes E, Tranter G, Nicholson JK, Bethell HW (2002). Rapid and non-invasive diagnosis of the presence and severity of coronary heart disease using 1H-NMR-based metabonomics. Nat Med.

[5] Casperson SL, Sheffield-Moore M, Hewlings SJ, Paddon-Jones D (2012). Leucine supplementation chronically improves muscle protein synthesis in older adults consuming the RDA for protein. Clin Nutr.

[6] Cederholm T, Wretlind B, Hellström K, Andersson B, Engström L, Brismar K (1997). Enhanced generation of interleukins 1 beta and 6 may contribute to the cachexia of chronic disease. Am J Clin Nutr.

[7] Coelho MG, Reis PA, Gava VB, Marques PR, Gayer CR, Laranja GA (2004). Anti-arthritic effect and subacute toxicological evaluation of Baccharis genistelloides aqueous extract. Toxicol Lett.

[8] Conway MW, Clark JF (1996). Creatine and creatine phosphate: scientific and clinical perspectives.

[9] Cuzzocrea S, McDonald MC, Mota-Filipe H, Mazzon E, Costantino G, Britti D (2000). Beneficial effects of tempol, a membrane-permeable radical scavenger, in a rodent model of collagen induced arthritis. Arth Rheum.

[10] Dawson JE, Salt HB (1952). Creatine metabolism in arthritis. Ann Rheum Dis.

[11] Ding X, Hu J, Li J, Zhang Y, Shui B, Ding Z (2014). Metabolomics analysis of collagen-induced arthritis in rats and interventional effects of oral tolerance. Anal Biochem.

[12] Fassbender HG, Simmling-Annefeld M (1983). The potential aggressiveness of synovial tissue in rheumatoid arthritis. J Pathol.

[13] Feldmann M (2001). Pathogenesis of arthritis: recent research progress. Nat Immunol.

[14] Feldmann M, Brennan FM, Maini RN (1996). Rheumatoid arthritis. Cell.

[15] Firestein GS (2003). Evolving concepts of rheumatoid arthritis. Nature.

[16] Gebregiworgis T, Powers R (2012). Application of NMR metabolomics to search for human disease biomarkers. Comb Chem High Throughput Screen.

[17] Gobelet C, Gerster JC (1984). Synovial fluid lactate levels in septic and non-septic arthritides. Ann Rheum Dis.

[18] Hawthorne JN, Ansell GB (1982). Phospholipids.

[19] Henderson B, Edwards JG, Pettipher ER (1995). Mechanisms and models in rheumatoid arthritis.

[20] Holmdahl R, Andersson ME, Goldschmidt TJ (1989). Collagen induced arthritis as an experimental model for rheumatoid arthritis. Immunogenetics, pathogenesis and autoimmunity. APMIS.

[21] Hwang GS, Yang JY, Ryu do H, Kwon TH (2010). Metabolic profiling of kidney and urine in rats with lithium-induced nephrogenic diabetes insipidus by (1) H-NMR-based metabonomics. Am J Physiol.

[22] Kannan K, Ortmann RA, Kimpel D (2005). Animal models of rheumatoid arthritis and their relevance to human disease. Pathophysiology.

[23] Kinne RW, Bräuer R, Stuhlmüller B, Palombo-Kinne E, Burmester GR (2000). Macrophages in rheumatoid arthritis. Arthritis Res.

[24] Kotler DP (2000). Cachexia. Ann Intern Med.

[25] Kumar N, Singh S, Patro N, Patro I (2009). Evaluation of protective efficacy of Spirulina platensis against collagen-induced arthritis in rats. Inflammopharmacology.

[26] Lazarevic MB, Vitic J, Mladenovic V, Myones BL, Skosey JL, Swedler WI (1992). Dyslipoproteinemia in the course of active rheumatoid arthritis. Semin Arthritis Rheum.

[27] Lee DM, Weinblatt ME (2001). Rheumatoid arthritis. Lancet.

[28] Marchesi JR, Holmes E, Khan F, Kochhar S, Scanlan P, Shanahan F (2007). Rapid and noninvasive metabonomic characterization of inflammatory bowel disease. J Proteome Res.

[29] Meister A (2012). Biochemistry of the amino acids.

[30] Meshitsuka S, Yamazaki E, Inoue M, Hagino H, Teshima R, Yamamoto K (1999). Nuclear magnetic resonance studies of synovial fluids from patients with rheumatoid arthritis and osteoarthritis. Clin Chim Acta.

[31] Nath RL (2007). A textbook of medicinal biochemistry.

[32] Naughton D, Whelan M, Smith EC, Williams R, Blake DR, Grootveld M (1993). An investigation of the abnormal metabolic status of synovial fluid from patients with rheumatoid arthritis by high field proton nuclear magnetic resonance spectroscopy. FEBS Lett.

[33] Naughton DP, Haywood R, Blake DR, Edmonds S, Hawkes GE, Grootveld M (1993). A comparative evaluation of the metabolic profiles of normal and inflammatory knee-joint synovial fluids by high resolution proton NMR spectroscopy. FEBS Lett.

[34] Nicholson JK, Lindon JC, Holmes E (1999). Metabonomics: understanding the metabolic responses of living systems to pathophysiological stimuli via multivariate statistical analysis of biological NMR spectroscopic data. Xenobiotica.

[35] Ohkawa H, Ohishi N, Yagi K (1979). Assay for lipid peroxides in animal tissues by thiobarbituric acid reaction. Anal Biochem.

[36] Park YB, Lee SK, Lee WK, Suh CH, Lee CW, Lee CH (1999). Lipid profiles in untreated patients with rheumatoid arthritis. J Rheumatol.

[37] Pasupathi P, Deepa M, Rani P, Sankar RR (2009). Circulating lipid peroxidation, plasma and erythrocyte antioxidant status in patients with rheumatoid arthritis. Bangladesh Med Res Counc Bull.

[38] Patro N, Sharma A, Kariaya K, Patro I (2011). Spirulina platensis protects neurons via suppression of glial activation and peripheral sensitization leading to restoration of motor function in collagen-induced arthritic rats. Indian J Exp Biol.

[39] Qin LQ, Xun P, Bujnowski D, Daviglus ML, Van Horn L, Stamler J (2011). (INTERMAP Cooperative Research Group). Higher branched-chain amino acid intake is associated with a lower prevalence of being overweight or obese in middle-aged East Asian and Western adults. J Nutr.

[40] Rajendram R, Preedy VR, Patel VB (2015). Branched chain amino acids in clinical nutrition, Vols 1-2.

[41] Reavley N (1998). The new encyclopaedia of vitamins, minerals, supplements & herbs: completely cross-referenced user’s guide for optimal health (p 374).

[42] Salvemini D, Mazzon E, Dugo L, Serraino I, De Sarro A, Caputi AP (2001). Amelioration of joint disease in a rat model of collagen-induced arthritis by M40403, a superoxide dismutase mimetic. Arthr Rheum.

[43] Shao W, Gu J, Huang C, Liu D, Huang H, Huang Z (2014). Malignancy-associated metabolic profiling of human glioma cell lines using 1H NMR spectroscopy. Mol Cancer.

[44] Sharma S, Sahu D, Das HR, Sharma D (2011). Amelioration of collagen-induced arthritis by Salix nigra bark extract via suppression of pro-inflammatory cytokines and oxidative stress. Food Chem Toxicol.

[45] Sharma U, Atri S, Sharma MC, Sarkar C, Jagannathan NR (2003). Biochemical characterization of muscle tissue of limb girdle muscular dystrophy: an 1H and 13C NMR study. NMR Biomed.

[46] Sharma U, Atri S, Sharma MC, Sarkar C, Jagannathan NR (2003). Skeletal muscle metabolism in Duchenne muscular dystrophy (DMD): an in-vitro proton NMR spectroscopy study. Magn Reson Imaging.

[47] Sinclair AJ, Viant MR, Ball AK, Burdon MA, Walker EA, Stewart PM (2010). NMR-based metabolomic analysis of cerebrospinal fluid and serum in neurological diseases - a diagnostic tool?. NMR Biomed.

[48] Singh RP, Padmavathi B, Rao AR (2000). Modulatory influence of Adhatoda vesica (Justicia adhatoda) leaf extract on the enzymes of xenobiotic metabolism, antioxidant status and lipid peroxidation in mice. Mol Cell Biochem.

[49] Sitton NG, Dixon JS, Bird HA, Wright V (1987). Serum biochemistry in rheumatoid arthritis, seronegative arthropathies, osteoarthritis, SLE and normal subjects. Br J Rheumatol.

[50] Srivastava NK, Pradhan S, Mittal B, Kumar R, Pandey CM, Gowda GA (2008). Novel corrective equations for complete estimation of human tissue lipids after their partial destruction by perchloric acid pre-treatment: high-resolution (1)H-NMR-based study. NMR Biomed.

[51] Srivastava NK, Sharma S, Purusottam RN, Sinha N, Singh R, Sharma D (2014). Abnormal lipid metabolism in collagen-induced arthritis rat model in vitro, high resolution NMR spectroscopy based analysis. Indian J Exp Biol.

[52] St. Clair EW, Pisetsky DS, Haynes BF (2004). Rheumatoid arthritis.

[53] Talwar GP, Srivastava LM (2006). Textbook of biochemistry and human biology.

[54] Tortora GT, Derrickson BH (2011). Principles of anatomy and physiology.

[55] Trang LE, Fürst P, Odebäck AC, Lövgren O (1985). Plasma amino acids in rheumatoid arthritis. Scand J Rheumatol.

[56] Trentham DE (1982). Collagen arthritis as a relevant model for rheumatoid arthritis. Arthr Rheum.

[57] Vance DE (1989). Phosphatidylcholine metabolism.

[58] Vijayakumar D, Suresh K, Manoharan S (2005). Altered pattern of lipids in plasma and erythrocyte membranes of rheumatoid arthritis patients. Indian J Clin Biochem.

[59] Weljie AM, Dowlatabadi R, Miller JB, Vogel HJ, Jirik FR (2007). An inflammatory arthritis-associated metabolite biomarker pattern revealed by 1H NMR spectroscopy. J Proteome Res.

[60] Young SP, Kapoor SR, Viant MR, Byrne JJ, Filer A, Buckley CD (2013). The impact of inflammation on metabolomic profiles in patients with arthritis. Arthr Rheum.

[61] Young SP, Nessim M, Falciani F, Trevino V, Banerjee SP, Scott RA (2009). Metabolomic analysis of human vitreous humor differentiates ocular inflammatory disease. Mol Vis.

[62] Yue R, Zhao L, Hu Y, Jiang P, Wang S, Xiang L (2013). Metabolomic study of collagen-induced arthritis in rats and the interventional effects of Huang-Lian-Jie-Du-Tang, a traditional chinese medicine. Evid Based Complement Alternat Med.

[63] Zhang H, Fu P, Ke B, Wang S, Li M, Han L (2014). Metabolomic analysis of biochemical changes in the plasma and urine of collagen-induced arthritis in rats after treatment with Huang-Lian-Jie-Du-Tang. J Ethnopharmacol.

[64] Zhong F, Liu X, Zhou Q, Hao X, Lu Y, Guo S (2012). 1H NMR spectroscopy analysis of metabolites in the kidneys provides new insight into pathophysiological mechanisms: applications for treatment with Cordyceps sinensis. Nephrol Dial Transplant.

